# Convergence of optimal expected utility for a sequence of binomial models

**DOI:** 10.1111/mafi.12326

**Published:** 2021-06-28

**Authors:** Friedrich Hubalek, Walter Schachermayer

**Affiliations:** ^1^ Research Unit of Financial and Actuarial Mathematics TU Wien Vienna Austria; ^2^ Faculty of Mathematics University of Vienna Vienna Austria

**Keywords:** Black‐Scholes‐Merton model, Cox‐Ross‐Rubinstein model, discrete versus continuous time, optimal expected utility

## Abstract

We consider the convergence of the solution of a discrete‐time utility maximization problem for a sequence of binomial models to the Black‐Scholes‐Merton model for general utility functions. In previous work by D. Kreps and the second named author a counter‐example for positively skewed non‐symmetric binomial models has been constructed, while the symmetric case was left as an open problem. In the present article we show that convergence holds for the symmetric case and for negatively skewed binomial models. The proof depends on some rather fine estimates of the tail behaviors of binomial random variables. We also review some general results on the convergence of discrete models to Black‐Scholes‐Merton as developed in a recent monograph by D. Kreps.

## 1 INTRODUCTION

Mark Davis has dedicated a large portion of his impressive scientific work to *Mathematical Finance*. He shaped this field by applying masterfully the tools from stochastic analysis which he dominated so well.

The present authors remember very well several discussions during Mark's 7 months stay in Vienna in 2000. Mark repeatedly expressed his amazement about the perfect match of Itô's stochastic calculus with the line of *Mathematical Finance* initiated by Black, Scholes, and Merton (Black and Scholes, 1973; Merton, 1973). In particular, Mark was astonished how well the martingale representation theorem fits to this theory and loved this connection. He also appreciated the approximation of the Black‐Scholes‐Merton model by binomial processes as initiated by Cox et al. (1979). The subtle notions of Itô integrals and the martingale representation theorem in continuous time boil down in the discrete setting to simple linear algebra as we all know from teaching *Mathematical Finance* to undergraduates.

Let us have a look back to the early days of *Mathematical Finance*. After the pioneering papers Black and Scholes (1973), Merton (1973), and Cox et al. (1979) the next step was taken by Harrison, Kreps, and Pliska in three articles Harrison and Kreps (1979), Kreps (1981), and Harrison and Pliska (1981), who paved the way from previous ad hoc arguments to a systematic study of the notions of arbitrage, martingale measures, martingale representation, complete markets, and the interconnections between these notions. This opened the arena where Mark made so many important contributions.

In the present note we want to go back to the roots and reconsider the approximation of the Black‐Scholes‐Merton model by discrete models such as the binomial model. Already Bachelier has viewed Brownian motion as an infinitesimal version of a symmetric random walk. This random walk view opens a very direct path from simple linear algebra to the martingale representation theorem. The guiding intuition is that a Brownian motion during each infinitesimal time interval has only two choices, namely going up or going down by a properly scaled infinitesimal.

But what happens if we take some other approximation of Brownian motion by discrete processes? The archetypical example is the “trinomial” model. In addition to the up‐ and down‐tick in the binomial model, there is a third intermediate possibility. In the limit you find the same (geometric) Brownian motion as for the binomial model. But if you try to apply the discrete time reasoning from the binomial case as in Cox et al. (1979) to the trinomial model, you immediately run into serious trouble. There is no replication argument available any more and the financial market becomes highly incomplete. The blunt reason is that you are looking for the solution of two linear equations with three unknowns so that there is no hope for a unique solution. As is well known, the unique arbitrage‐free option prices of the binomial models are replaced by an interval of arbitrage‐free prices in the trinomial model, whose lower and upper bounds are given by the sub‐ and super‐replication prices. Typically these intervals become very wide and are of no practical relevance.

But is this really the last word? From an economic point of view this sharp distinction between two similar approximations of the same object seems to be artificial. Can one find a more satisfactory answer? This question recently triggered the attention of David Kreps and led him to take up the theme of option pricing again, where he had made fundamental contributions some 40 years ago. This renewed interest resulted in the monograph Kreps (2019) which appeared in 2019. Kreps' starting point was a simulation of the results of delta‐hedging in the framework of a trinomial model. He applied this rather naive strategy to a standard European option and plotted the outcomes of 500 simulations (see figure 1.1 of Kreps (2019)).The result was amazing: With bare eyes one can hardly see the difference between the precise terminal option value and the result of the delta hedge. The visual impression of the outcome of the simulations is that of a complete market.

To analyze this phenomenon in proper generality let us fix some notation as in Kreps' monograph (Kreps 2019). We work in the space $\Omega =C_{0}\left[0,1\right]$, the space of all continuous functions ω from [0,1] to R whose value at 0 is 0. We let ω denote a typical element of Ω, with ω(t) the value of ω at date t. Let P be Wiener measure on Ω.

We consider a Black‐Scholes‐Merton model of the form $S\left(t,\omega \right)=e^{\omega (t)}$ for the stock, taking the bond as numeraire. We know that there is a unique probability measure on Ω, denoted $P^{*}$, that is equivalent to P and, under which, S(t) is a martingale (Harrison and Kreps (1979)).

Contingent claims are Borel‐measurable functions x:Ω→R. We let X denote the space of bounded and contingent claims which are continuous with respect to the norm topology on $\Omega =C_{0}\left[0,1\right]$. The well‐known “complete markets” result for the Black‐Scholes model says that, for every x∈X, x can uniquely be written

$$x=E_{P^{*}}\left[x\right]+\int_{0}^{1}\alpha dS,$$

for a predictable and S‐integrable integrand α.

Now suppose that for n=1,2,…, we have different probability measures $P_{n}$ defined on Ω, with the following structure: For each n, the support of $P_{n}$ consists of piecewise linear functions that, in particular, are linear on all intervals of the form [k/n,(k+1)/n], for k=0,…,n−1. The interpretation is that $P_{n}$ represents a probability distribution on paths of the log of the stock price in an n‐th discrete‐time economy, in which trading between the stock and bond is possible only at times t=k/n for k=0,…,n−1. At time 1, the bond and stock liquidate in state ω at prices 1 and $e^{\omega (1)}$.

Consumers in the n‐th discrete‐time economy can implement (state‐dependent) self‐financing trading strategies $\left(V\left(0\right),\left\{\alpha^{n}\left(k/n\right),k=0,\ldots ,n-1\right\}\right)$, where the interpretation is that V(0) is the value of the consumer's initial portfolio, $\alpha^{n}\left(k/n,\omega \right)$ is the number of shares of stock held by the consumer *after* she has traded at time k/n, and, after time 0, bond holdings are adjusted that any adjustments in stock holdings at times k/n are financed with bond purchases/sales. In the n‐th economy, the consumer only knows at time k/n the evolution of the stock price up to and including that date. In the usual fashion, if V(k/n,ω) is the value of the portfolio formed by this trading strategy at time k/n in state ω, then for all k=1,…,n,

$$V\left(k/n,\omega \right)=V\left(0\right)+\sum_{j=1}^{k-1}\alpha^{n}\left(j/n,\omega \right)\left[S\left((j+1)/n,\omega \right)-S\left(j/n,\omega \right)\right].$$

We maintain throughout the assumption that, for each n, $P_{n}$ specifies an arbitrage‐free model of a financial market in the usual sense: It is impossible to find in the n‐th discrete‐time model a trading strategy $\left(V\left(0\right),\alpha^{n}\right)$ with V(0)=0, V(1)≥0 $P_{n}$‐a.s., and V(1)>0 with $P_{n}$‐positive probability. This is true if and only if there exists a probability measure $P_{n}^{*}$ that is equivalent to $P_{n}$, under which $\left\{\left(e^{\omega (k/n)},F_{k/n}\right);k=0,\ldots ,n\right\}$ is a martingale (Dalang, Morton, Willinger Dalang et al. (1990)). Such a $P_{n}^{*}$ is called an *equivalent martingale measure* (emm) for the n‐th discrete‐time model. Of course, in general there will be more than one emm $P_{n}^{*}$. However, with respect to any emm $P_{n}^{*}$, $\left(V\left(k/n\right),F_{k/n}\right)$ is a martingale with respect to $P_{n}^{*}$. In particular, the expectation of V(1) under every emm $P_{n}^{*}$ is V(0).

Let $X^{n}:=\{x\in X:x\left(\omega \right)=V\left(1,\omega \right)$ for some trading strategy $\left(V\left(0\right),\alpha^{n}\right)$ for the nth discrete‐time economy}. We refer to $X^{n}$ as the space of synthesizable claims in the n‐th discrete‐time economy.

A basic question treated in detail in Kreps' mongraph Kreps (2019) is the following: in which precise sense and under which precise assumptions can elements of X be approximated by elements of $X^{n}$? During a visit of the second named author to Stanford University in the spring term 2019 we jointly took up this scheme in the paper Kreps and Schachermayer (2021) and found the following definition to be suitable.

Definition 1.1 The claim x can be *asymptotically synthesized with* x *‐controlled risk* if, for every ε>0, there exists $N_{\varepsilon }$ such that, for all $n>N_{\varepsilon }$, there is $x^{n}\in X^{n}$ with

$$P_{n}\left(\left\{\omega :\right|x^{n}\left(\omega \right)-x\left(\omega \right)|>\varepsilon \}\right)<\varepsilon ,$$

and, in addition, $P_{n}\left(\left\{\omega :\underline{x}\leq x^{n}\left(\omega \right)\leq \bar{x}\right\}\right)=1$ for all n, where $\underline{x}=\inf_{\omega }x\left(\omega \right)$ and $\bar{x}=\sup_{\omega }x\left(\omega \right)$.

The main result result of Kreps and Schachermayer (2020) states that, under mild conditions which are natural in the present context, x‐controlled risk can be attained:

Theorem 1.2 [KS21,Theorem 1] Suppose that the probability measures $P_{n}$ on $\Omega =C_{0}\left[0,1\right]$ weakly tend to Wiener measure P, and that, for some sequence $\left\{\delta_{n};n=1,\ldots ,\right\}$ of positive numbers tending to zero,

(1) $$P_{n}\left(\{\omega :\underset{0\leq k<n}{\sup}\left|\omega \left(k/n\right)-\omega \left(\left(k+1\right)/n\right)\right|\leq \delta_{n}\}\right)=1.$$

Then every (continuous and bounded) x∈X can be asymptotically synthesized with x‐controlled risk. Moreover, fixing the claim x, the sequence of claims $\left\{x^{n}\right\}$ that asymptotically synthesize x can be chosen where, for $\left(V^{n}\left(0\right),\alpha^{n}\right)$ the trading strategy that gives $x^{n}$, $V^{n}\left(0\right)\equiv E_{P^{*}}\left[x\right]$, the Black‐Scholes‐Merton price of the claim x.

As a particular example, the theorem applies, for example, to the trinomial model and, more generally, to a wide range of incomplete approximations of the Black‐Scholes model. The message is: replacing the notions of sub‐ and super‐replications by Definition 1.1 we obtain economically meaningful notions of synthesis also in incomplete markets. This is in contrast to the no‐arbitrage bounds which typically only yield huge intervals.

Theorem 1.2 settles the issue of replication of contingent claims. However, this result immediately triggers the next question: what about utility maximization when passing from a discrete approximation to the limiting Black‐Scholes‐Merton model? This question too is amply discussed in Kreps' monograph Kreps (2019) and was further pursued in another paper Kreps and Schachermayer (2020) by Kreps and the second named author.

Let us recapitulate the setting which is slightly more structured than the assumption of Theorem 1.2 above.

Fix a random variable ζ with mean zero, variance one, and bounded support. For an i.i.d. sequence $\left\{\zeta_{j};j=1,2,\ldots \right\}$, where each $\zeta_{k}$ has the distribution of ζ, the law for the price of the stock at time k/n is

(2) $$S\left(k/n\right):=e^{\xi (k/n)}\;\;\text{where}\;\;\xi \left(k/n\right):=\sum_{j=1}^{k}\frac{\zeta_{j}}{\sqrt{n}}.$$

Again we embed this model into the standard state space $\Omega =C_{0}\left[0,1\right]$. For each n, let $P_{n}$ be the probability measure on Ω such that the joint distribution of (ω(0),ω(1/n),…,ω(1)) matches the distribution of (ξ(0),ξ(1/n),…,ξ(1)), and such that ω(t) for k/n<t<(k+1)/n is the linear interpolate of ω(k/n) and ω((k+1)/n). And let $S:\Omega \to R_{+}$ be defined by $S\left(\omega ,t\right)=e^{\omega (t)}$. Donsker's Theorem tells us that $P_{n}$ weakly tends to P, where P is Wiener measure on $C_{0}\left[0,1\right]$.

We imagine an expected‐utility‐maximizing agent who is endowed with initial wealth x, trading as above either in the discrete market or in the continuous limit.

The question addressed in Kreps (2019) is: If we place this consumer in the nth discrete‐time economy (where the stock and bond trade (only) at times 0,1/n,2/n,…,(n−1)/n), does the optimal expected utility she can attain approach, as n→∞, what she can optimally attain in the continuous‐time Black‐Scholes‐Merton economy?

Let $u_{n}\left(x\right)$ be the supremal expected utility she can attain in the nth discrete‐time economy if her initial wealth is x, and let u(x) be her supremal expected utility in the Black‐Scholes‐Merton economy. Kreps (2019) obtained partial one‐sided results, showing that $\lim\inf_{n}u_{n}\left(x\right)\geq u\left(x\right)$. And he proved $\lim_{n}u_{n}\left(x\right)=u\left(x\right)$ in the very special cases of U having either constant absolute or relative risk aversion. But he only conjectures that the second “half”, or $\lim\sup_{n}u_{n}\left(x\right)\leq u\left(x\right)$ is true for general (sufficiently regular) U.

To tackle this issue in proper generality we first need precise definitions

Definition 1.3 A utility function U is a strictly increasing, strictly concave, and continuously differentiable function $U:R_{+}\to R$, which satisfies the Inada conditions that $\lim_{x\to 0}U^{\prime }\left(x\right)=\infty$ and $\lim_{x\to \infty }U^{\prime }\left(x\right)=0$.

As usual, we define the corresponding value functions $u_{n}\left(x\right)$ and u(x) as the maximal expected utility an agent can achieve from initial wealth x by admissibly trading in the markets defined by the measures $P_{n}$ and P.

For the utility function U, its asymptotic elasticity Kramkov and Schachermayer (1999), written AE(U), is defined by

$$\text{AE}\left(U\right):=\lim\underset{x\to \infty }{\sup}\frac{xU^{\prime }\left(x\right)}{U(x)}$$

If, for instance, $U\left(x\right)=x^{\alpha }/\alpha$ for α∈(0,1), then AE(U)=α.

The concavity of U implies that AE(U)≤1 in all cases; if U is bounded above and if U(∞)>0, then AE(U)=0. But if U(∞)=∞, AE(U) can equal 1; an example is where U(x)=x/ln(x) for sufficiently large x.

The following theorem gives an affirmative answer to Kreps' conjecture under the asymptotic elasticity condition.

Theorem 1.4 [KS 20 Theorem 1] Suppose that the utility function U satisfies AE(U)<1. Then, for all x>0, the value function x↦u(x) is finite‐valued and

(3) $$\lim_{n\to \infty }u_{n}\left(x\right)=u\left(x\right).$$

To resume, *admitting the condition* AE(U)<1, this theorem settles the issue of convergence of the optimal expected utility in the discrete approximations of the Black‐Scholes‐Merton model in an economically satisfactory way. Note that we did *not* suppose the completeness of the discrete markets modeled by the measures $P_{n}$. In other words: the convergence of expected utility behaves well, independently of whether we are in the binomial or in the trinomial approximation. Also note that the assertion of finiteness of both terms in (3)—as a consequence of the asymptotic elasticity assumption—is a non‐trivial result.

But, of course, at this stage the next question pops up. What happens for the—admittedly somewhat pathological—case of utility functions with AE(U)=1? For this case Kreps and the second named author found to their surprise that the answer to Kreps' conjecture turns out to be negative. More surprisingly: this pathology already happens in the framework of the binomial model!

To address this issue let us fix the notation for the special case of the binomial model in (2).

For arbitrary p∈(0,1) we consider and i.i.d. sequence ${\left(\alpha_{n}\right)}_{n=1}^{\infty }$ of Bernoulli variables with

(4) $$P\left[\alpha_{n}=0\right]=1-p,\;P\left[\alpha_{n}=1\right]=p,$$

where p∈(0,1). Denote by $\zeta_{n}$ the corresponding standardized variables

(5) $$\zeta_{n}=\frac{\alpha_{n}-p}{\sqrt{p(1-p)}},$$

so that $E[\zeta_{n}]=0$ and $\mathrm{Var}[\zeta_{n}]=1$. Again we denote by $\xi_{n,k}$ the scaled partial sums

(6) $$\xi_{n,k}=\frac{1}{\sqrt{n}}\sum_{j=1}^{k}\zeta_{j}$$

and set

(7) zn,k=k−npnp(1−p),fn,k=nkpk(1−p)n−k,k=0,…,n.

Then $f_{n,k}=P\left[\xi_{n,n}=z_{n,k}\right]$.

If we again set $S^{\left(n\right)}\left(k/n\right)=e^{\xi_{n,k}}$ and extend $S^{\left(n\right)}$ by interpolation to continuous‐time processes as above, then ${\left(S^{\left(n\right)}\left(t\right)\right)}_{0\leq t\leq 1}$ approximates the Black‐Scholes‐Merton model ${\left(S\left(t\right)\right)}_{0\leq t\leq 1}$ with S(t)=exp(ω(t)) for 0≤t≤1.

The distribution of the random variable ω(1) under $P_{n}$ equals the binomial distribution of $\xi_{n,n}=\frac{\zeta_{1}+\cdots +\zeta_{n}}{n^{\frac{1}{2}}}$.

Again we define the value functions u(x) and $u_{n}\left(x\right)$ as

(8) $$u\left(x\right)=\sup\left\{E_{P}\left[U(X)\right]:E_{P^{*}}\left[X\right]\leq x\right\}$$

and

(9) $$u_{n}\left(x\right)=\sup\left\{E_{P_{n}}\left[U(X_{n})\right]:E_{P_{n}^{*}}\left[X_{n}\right]\leq x\right\},$$

where $P^{*}$ and $P_{n}^{*}$ now are the unique equivalent martingale measures pertaining to the Black‐Scholes‐Merton model and its n‐th approximation, respectively.

When p∈(0,1/2) we have $E[\zeta_{n}^{3}]>0$. This is the case where things go astray, as demonstrated by the counterexample in Section 9 of Kreps and Schachermayer (2020). If p∈(0,1/2), there is a utility function U satisfying the conditions of Definition 1.3 (but with AE(U)=1) such that u(x) is a perfectly well‐behaved finite function while $\lim_{n\to \infty }u_{n}\left(x\right)=\infty$, for all x>0. This phenomenon happens if $E[\zeta_{n}^{3}]>0$ which means that the up‐tick of the log‐price is larger than the down‐tick.

It was left as an open question in Kreps and Schachermayer (2020) what happens in the case $E[\zeta_{n}^{3}]\leq 0$ with special emphasis on the symmetric case $E[\zeta_{n}^{3}]=0$ when the up‐tick of the log‐price is equal to the down‐tick.

The good news is that in this case everything works out as it should as stated in the subsequent theorem which is the main novel contribution of the present paper.

Theorem 1.5 If U and ζ are as above with p∈[1/2,1), we have

(10) $$u\left(x\right)=\lim_{n\to \infty }u_{n}\left(x\right),\;x>0.$$

The theorem will follow from the subsequent more technical version of (10). As above, let

$$u\left(x\right):=\sup\{E_{P}\left[U\left(X\right)\right]:E_{P^{*}}\left[X\right]\leq x\},$$

and

$$u_{n}\left(x\right):=\sup\{E_{P_{n}}\left[U\left(X\right)\right]:E_{P_{n}^{*}}\left[X\right]\leq x\},$$

where $P_{n}^{*}$ and $P^{*}$ denote the unique equivalent martingale measures of the binomial and the Black‐Scholes‐Merton model, respectively.

As usual we denote by $V:R_{+}\to R$ the conjugate function of U, that is, $V\left(y\right)=\sup_{x>0}\left\{U\left(x\right)-xy\right\}$, and the corresponding dual value functions by

$$v\left(y\right):=E_{P}\left[V\left(y\frac{dP^{*}}{dP}\right)\right],$$

and

$$v_{n}\left(y\right):=E_{P_{n}}\left[V\left(y\frac{dP_{n}^{*}}{dP_{n}}\right)\right].$$

Proposition 1.6 Under the assumptions of Theorem 1.5 we have

(11) $$\underset{n\to \infty }{\mathrm{lim sup}}v_{n}\left(y\right)\leq v\left(y\right),\;y>0.$$

Proof of Theorem 1.5 (admitting Proposition 1.6) We deduce from (Kreps and Schachermayer, 2020, Proposition 2) and standard results on conjugate functions that the reverse inequality to (11) does hold true, that is,

(12) $$\underset{n\to \infty }{\mathrm{lim inf}}v_{n}\left(y\right)\geq v\left(y\right),\;y>0.$$

Admitting Proposition 1.6, formulas (11) and (12) imply the equality

(13) $$\lim_{n\to \infty }v_{n}\left(y\right)=v\left(y\right),\;y>0.$$

Using again standard results on conjugate functions (compare Kreps and Schachermayer (2020)) we obtain (10) from (13).□

We therefore are left to show Proposition 1.6 which will be a technically demanding task. A key ingredient for the proof of Proposition 1.6 are estimates for the tails of the standardized binomial distributions in terms of the standard Gaussian tails.

Let ξ be a standard normal random variable and denote its density by $\phi \left(x\right)=\frac{1}{\sqrt{2\pi }}e^{-\frac{x^{2}}{2}}$. Set

(14) $$F_{n}\left(x\right)=P\left[\xi_{n,n}\leq x\right],\;\Phi \left(x\right)=P\left[\xi_{\leq }x\right]$$

and

(15) $${\bar{F}}_{n}\left(x\right)=1-F_{n}\left(x\right)=P\left[\xi_{n,n}>x\right],\;\bar{\Phi }\left(x\right)=1-\Phi \left(x\right)=P\left[\xi >x\right].$$

Proposition 1.7 Suppose p∈[1/2,1), then there is C>0 such that, for n≥1, we have

(16) $$f_{n,k}\leq C\cdot \frac{1}{\sqrt{np(1-p)}}\phi \left(z_{n,k-1}\right),\;0\leq k\leq \left\lceil np\right\rceil ,$$

(17) $$f_{n,k}\leq C\cdot \frac{1}{\sqrt{np(1-p)}}\phi \left(z_{n,k+1}\right),\;\left\lfloor np\right\rfloor \leq k\leq n.$$

Furthermore

(18) $$F_{n}\left(x\right)\leq C\Phi \left(x\right),\;x\leq 0$$

and

(19) $${\bar{F}}_{n}\left(x\right)\leq C\bar{\Phi }\left(x\right),\;x\geq 0.$$

Remark 1.8 The terms $\frac{1}{\sqrt{np(1-p)}}\phi \left(z_{n,k\pm 1}\right)$ in (16) and (17) are a lower bound for the area under the density φ(x) between $z_{n,k-1}$ and $z_{n,k}$ on the left and $z_{n,k}$ and $z_{n,k+1}$ on the right tail, respectively.

We prove Proposition 1.6 and Proposition 1.7 first for the symmetric case p=1/2 in Section 2, as this case allows for several simplifications and the main arguments are more transparent. We then provide the slightly more technical details for the asymmetric case p∈(1/2,1) in Section 3.

Remark 1.9 Of course the history of the Central Limit Theorem goes back to prehistoric times. William Feller said in 1945 in his article Feller (1945):

Although the problem of an efficient estimation of the error in the normal approximation to the binomial distribution is classical, the many papers which are still being written on the subject show that not all pertinent questions have found a satisfactory solution.

We believe his statement remains valid to the present day, below some recent publications on that topic are given below and in the references. Further on Feller says:

What is really needed in many applications is an estimate of the relative error, but this seems difficult to obtain.

Here the control of the relative error is crucial for our application to utility maximization.

For small values of k, namely

(20) $$\frac{n}{2}<k\leq \frac{n}{2}+c\sqrt{n}$$

Proposition 1.7 follows from an old and well‐known limit theorem from the proof of the De Moivre‐Laplace Central Limit Theorem, see, for example, (Feller, 1968, Theorem VII.3.1, p. 184) and set $p=q=\frac{1}{2}$ and $K_{n}=c\sqrt{n}$. Serov and Zubkov in 2013 remark in their article Zubkov and Serov (2013):

But relative errors of the Moivre‐Laplace approximations for the tails of binomial distribution function are large.

Based on a corollary of a theorem given by Chernoff, (Okamoto, 1958, Theorem 1.i, p. 33) yields for p=1/2, in our notation, the inequality ${\bar{F}}_{n}\left(x\right)<\sqrt{2\pi }\phi \left(x\right)$. Since $\bar{\Phi }\left(x\right)∼x^{-1}\phi \left(x\right)$ as x→∞ our estimate improves asymptotically by a factor of 1/x as x→∞. This is important for the utility application.

In (Desolneux et al., 2008, Chapter 4) we find an impressive discussion of a large list of inequalities for the binomial distribution.

## 2 THE SYMMETRIC CASE

Proof of Proposition 1.6 ((symmetric case) admitting Proposition 1.7) As in (Kreps and Schachermayer, 2020, Section 3) we write

(21) $$v\left(y\right)=E_{P}\left[V\left(yZ\right)\right],\;y>0,$$

and

(22) $$v_{n}\left(y\right)=E_{P_{n}}\left[V\left(yZ_{n}\right)\right],\;y>0,$$

where V(y)=sup{U(x)−xy:x>0} is the conjugate function of U.

The random variables Z and $Z_{n}$ are the densities of the (unique) equivalent martingale measures $P^{*}$ and $P_{n}^{*}$ with respect to P and $P_{n}$, respectively, that is, $Z=\frac{dP^{*}}{dP}$ and $Z_{n}=\frac{dP_{n}^{*}}{dP_{n}}$. They are of the form

(23) $$Z=\exp\left(-\frac{\omega (1)}{2}-\frac{1}{8}\right)$$

and

(24) $$Z_{n}=\exp\left(-a_{n}\omega \left(1\right)-b_{n}\right).$$

In the symmetric case the calculations from (Kreps and Schachermayer, 2020, Section 6) simplify, and we have that

(25) $$a_{n}=\frac{1}{2},\;b_{n}=n\log\cosh\left(\frac{1}{2\sqrt{n}}\right).$$

It follows that $b_{n}$ increases to 1/8 as n→∞.

Fix y>0 such that v(y)<∞, otherwise (11) is certainly true. Denote by $H_{y}:R\to R$ the function

(26) $$H_{y}\left(x\right)=V\left(y\exp\left(-\frac{x}{2}-\frac{1}{8}\right)\right),\;x\in R,$$

and by $H_{y}^{n}:R\to R$ the function

(27) $$H_{y}^{n}\left(x\right)=V\left(y\exp\left(-\frac{x}{2}-b_{n}\right)\right),\;x\in R.$$

Clearly, these functions are increasing on R. Note, however, that they are not necessarily concave. We know that

(28) $$\begin{array}{cc} v(y) & =E_{P}\left[H_{y}\left(\omega \left(1\right)\right)\right]=\int_{R}H_{y}\left(x\right)\phi \left(x\right)dx<\infty \end{array}$$

while

(29) $$\begin{array}{cc} v_{n}\left(y\right) & =E_{P_{n}}\left[H_{y}^{n}\left(\omega \left(1\right)\right)\right]=\sum_{k=0}^{n}H_{y}^{n}\left(z_{n,k}\right)f_{n,k}, \end{array}$$

where ϕ(x) is the standard normal density and $f_{n,k}$ are the binomial probabilities as in Section 1.

As $H_{y}^{n}\left(x\right)\leq H_{y}\left(x\right)$ for all x∈R, in order to show (11), it will suffice to show

(30) $$\underset{n\to \infty }{\mathrm{lim sup}}E_{P_{n}}\left[H_{y}\left(\omega \left(1\right)\right)\right]\leq E_{P}\left[H_{y}\left(\omega \left(1\right)\right)\right].$$

In order to show (30) the crucial estimate is the uniform integrability of the random variables $H_{y}\left(\omega \left(1\right)\right)$ under $P_{n}$. More precisely, we need the following estimates (31) and (32). For ε>0 there is M>0 such that

(31) $$E_{P_{n}}\left[H_{y}\left(\omega \left(1\right)\right)1_{\left\{H_{y}\left(\omega \left(1\right)\right)>M\right\}}\right]=\sum_{k=0}^{n}H_{y}\left(z_{n,k}\right)1_{\left\{H_{y}\left(z_{n,k}\right)>M\right\}}f_{n,k}<\varepsilon ,$$

and

(32) $$E_{P_{n}}\left[|H_{y}\left(\omega \left(1\right)\right)|1_{\left\{H_{y}\left(\omega \left(1\right)\right)<-M\right\}}\right]=\sum_{k=0}^{n}\left|H_{y}\left(z_{n,k}\right)\right|1_{\left\{H_{y}\left(z_{n,k}\right)<-M\right\}}f_{n,k}<\varepsilon ,$$

uniformly in n∈N. Formulas (31) and (32) correspond to the formulas (Kreps and Schachermayer, 2020, (8.12) and (8.14)).

First we consider $M>H_{y}{\left(1\right)}_{+}$. If $H_{y}\left(z_{n,k}\right)>M$, then n/2<k≤n and we are in a position to invoke formula (17) of Theorem 1.7, which gives an estimate on the right tail of the binomial distribution as compared to the normal one. More precisely, there is a universal constant C>0 such that, for every n∈N and n/2<k≤n and all $x\in (z_{n,k},z_{n,k+1})$

(33) $$f_{n,k}\leq C\cdot \frac{2}{\sqrt{n}}\phi \left(x\right),\;0\leq H_{y}\left(z_{n,k}\right)\leq H_{y}\left(x\right).$$

Thus

(34) $$\sum_{k=0}^{n}H_{y}\left(z_{n,k}\right)I_{\left\{H_{y}\left(z_{n,k}\right)>M\right\}}f_{n,k}\leq C\int_{-\infty }^{+\infty }H_{y}\left(x\right)I_{\left\{H_{y}\left(x\right)>M\right\}}\phi \left(x\right)dx.$$

It follows from (28) that the right‐hand side of (34) can be made smaller than ε for sufficiently large M.

A similar estimate applies for the left tail. We consider now $M>H_{y}{\left(-1\right)}_{-}$. If $H_{y}\left(z_{n,k}\right)<-M$ then 0≤k<n/2 and we invoke formula (16) of Theorem 1.7. We get now for every n∈N and 0≤k<n/2 and all $x\in (z_{n,k-1},z_{n,k})$

(35) $$f_{n,k}\leq C\cdot \frac{2}{\sqrt{n}}\phi \left(x\right),\;H_{y}\left(x\right)\leq H_{y}\left(z_{n,k}\right)\leq 0.$$

Thus

(36) $$\sum_{k=0}^{n}|H_{y}\left(z_{n,k}\right)|I_{\left\{H_{y}\left(z_{n,k}\right)<-M\right\}}f_{n,k}\leq C\int_{-\infty }^{+\infty }\left|H_{y}\left(x\right)\right|I_{\left\{H_{y}\left(x\right)<-M\right\}}\phi \left(x\right)dx.$$

Again, it follows from (28) that the right‐hand side of (36) can be made smaller than ε for sufficiently large M.

Using the well‐known weak convergence of $P_{n}$ to P and the uniform integrability conditions we can deduce (29), see (van der Vaart, 1998, Thm 2.20, p. 17).

Finally we consider the case $y=y_{0}$, where $y_{0}=\inf\left\{y>0:v\left(y\right)<\infty \right\}$, for the case $y_{0}>0$. Either $v(y_{0})=\infty$ in which case (11) holds trivially. Or $v(y_{0})<\infty$, in which case v is right continuous at $y_{0}$, see Kramkov and Schachermayer (1999); we therefore may repeat the above argument with $y=y_{0}$.

This finishes the proof of Proposition 1.6.□

Proof of Proposition 1.7 (symmetric case) Let us start with (17). It is enough to prove that there is $n_{0}>0$ such that (17) holds for $n\geq n_{0}$, since ϕ is strictly positive and there are only finitely many remaining cases that can be incorporated in the value of the constant C.

Let us consider first the extreme case k=n. In this case (17) follows since $p_{n,n}=2^{-n}$, which decays faster than $n^{-1/2}\phi \left(z_{n,n+1}\right)\approx e^{-\frac{n}{2}}$ for n→∞, as log2>1/2. Here we used the convention $z_{n,n+1}=\sqrt{n}+2/\sqrt{n}$, although $z_{n,n+1}$ is not a possible value for $Y_{n}$. Passing to the other extreme case, we deduce from the central limit theorem, that for k=⌊n/2⌋ we have $\frac{\sqrt{n}f_{n,k}}{2\phi (z_{n,k+1})}\to 1$ as n→∞.

For the remaining cases, ithat is, ⌊n/2⌋<k≤n−1, we take logarithms and show that

(37) $$\log\left(\frac{\sqrt{n}f_{n,k}}{2\phi (z_{n,k+1})}\right)$$

is bounded from above. To estimate the numerator in (37) we use a fine version of Stirling's Formula as given in (Abramowitz and Stegun, 1992, 6.1.38, p. 257), namely

(38) $$x!=\sqrt{2\pi }x^{x+\frac{1}{2}}\exp\left(-x+\frac{\theta (x)}{12x}\right),\;x>0,$$

with 0<θ(x)<1 for all x>0. We also note that $\lim_{x\to \infty }\theta \left(x\right)=0$. We obtain the estimates

(39) $$\begin{array}{ccc} \log(n!) & \leq & \log\sqrt{2\pi }+\left(n+\frac{1}{2}\right)\log n-n+\frac{1}{12n} \end{array}$$

(40) $$\begin{array}{ccc} \log(k!) & \geq & \log\sqrt{2\pi }+\left(k+\frac{1}{2}\right)\log k-k \end{array}$$

(41) $$\begin{array}{ccc} \log((n-k)!) & \geq & \log\sqrt{2\pi }+\left(n-k+\frac{1}{2}\right)\log\left(n-k\right)-\left(n-k\right). \end{array}$$

This yields an upper bound for the numerator of (37), as

(42) $$\log f_{n,k}=\log\left(n!\right)-\log\left(k!\right)-\log\left(\left(n-k\right)!\right)-n\log2.$$

As regards the denominator of (37), we have

(43) $$\log\phi \left(z_{n,k+1}\right)=2+2k-\frac{2}{n}-4\frac{k}{n}-2\frac{k^{2}}{n}-\frac{n}{2}-\log\sqrt{2\pi }.$$

Writing logk=logn+log(k/n) and log(n−k)=logn+log(1−k/n) and combining (42), (39), (40), (41), and (43) yields

(44) $$\log\left(\frac{\sqrt{n}f_{n,k}}{2\phi (z_{n,k+1})}\right)\leq g_{n}\left(\frac{k}{n}\right),$$

with $g_{n}\left(w\right)=\alpha \left(w\right)n+\beta_{n}\left(w\right)$, where $w\in [\frac{1}{2},1]$, and

(45) $$\alpha \left(w\right)=-w\log w-\left(1-w\right)\log\left(1-w\right)-2w\left(1-w\right)+\frac{1}{2}-\log2$$

and

(46) $$\beta_{n}\left(w\right)=-\frac{1}{2}\log w-\frac{1}{2}\log\left(1-w\right)+4w-2+\frac{25}{12n}.$$

It remains to show that $g_{n}\left(w\right)$ is bounded from above uniformly in n∈N and w∈[1/2,1−1/n].

We have $\alpha \left(\frac{1}{2}\right)=\alpha^{\prime }\left(\frac{1}{2}\right)=\alpha^{\prime \prime }\left(\frac{1}{2}\right)=\alpha^{\prime \prime \prime }\left(\frac{1}{2}\right)=0$ and for the forth derivative we have $\alpha^{iv}\left(w\right)=-2/{\left(1-w\right)}^{3}-2/w^{3}<0$ for $w\in (\frac{1}{2},1)$, and thus each of the functions $\alpha^{\prime \prime \prime }\left(w\right)$, $\alpha^{\prime \prime }\left(w\right)$, $\alpha^{\prime }\left(w\right)$, and α(w) is strictly negative and decreasing for $w\in (\frac{1}{2},1)$.

We have $\beta_{n}\left(1/2\right)=25/\left(12n\right)$ and $\beta_{n}^{\prime }\left(w\right)=4-1/\left(2w\right)+1/\left(2\left(1-w\right)\right)>0$ for $w\in (\frac{1}{2},1)$, thus $\beta_{n}\left(w\right)$ is strictly positive and strictly increasing for $w\in (\frac{1}{2},1)$.

For w∈[1/2,3/4] we have $g_{n}\left(w\right)\leq b_{n}\left(3/4\right)$. As $\lim_{n\to \infty }\beta_{n}\left(3/4\right)=1+\log\left(2/\sqrt{3}\right)<\infty$ it follows that $g_{n}\left(w\right)$ is bounded from above for the interval under consideration.

For w∈[3/4,1−1/n] we have $g_{n}\left(w\right)\leq \alpha \left(3/4\right)n+\beta_{n}\left(1-1/n\right)$. Now α(3/4)<0 and $\beta_{n}\left(1-1/n\right)∼\frac{1}{2}\log n$ as n→∞. Here the second term on the right hand side of (46) is the leading term. Finally we use the fact that α(3/4)n grows quicker than $\frac{1}{2}\log n$ to conclude that $g_{n}\left(w\right)$ is negative for w∈[3/4,1−1/n] and sufficiently large n.

The proof of (16) is completely symmetric with $g_{n}\left(w\right)$ for w∈[1/2,1−1/n] replaced by $g_{n}\left(1-w\right)$ for w∈[1/n,1/2].

Having proved (16) and (17) we mentioned already in Remark 1.8 how these two inequalities imply  (18) and (19).□

For the above proof of Proposition 1.6 the estimates (16) and (17) involving an unspecified constant C>0 is sufficiently strong. But we we can do better than that. We may adapt the above argument to yield a constant C=1+ε for n sufficiently large. Indeed, analyzing the above proof of Proposition 1.7, w e see that the above argument also works when we split the interval $\left(\frac{1}{2},1\right)$ not at w=3/4, but at a point θ∈(1/2,1), which is close to 1/2 to obtain a better constant C, for large enough n. The detailed argument is given in the proof of the following proposition, which sharpens Proposition 1.7.

Proposition 2.1 For any C>1 there is $n_{0}\left(C\right)>0$ such that equations (16)–(17) and (18)–(19) hold for $n\geq n_{0}\left(C\right)$.

We consider $\vartheta \in (\frac{1}{2},1)$ and proceed as in the proof of the Proposition 1.7 above. We distinguish two cases, $w\in [\frac{1}{2},\vartheta ]$ and w∈[ϑ,1−1/n]. In the first case, when w∈[1/2,ϑ], we have $g_{n}\left(w\right)\leq \beta_{n}\left(\vartheta \right)$ and

(47) $$\lim_{n\to \infty }\beta_{n}\left(\vartheta \right)=-\frac{1}{2}\log\vartheta -\frac{1}{2}\log\left(1-\vartheta \right)+4\vartheta -2-\log2.$$

The right hand side is increasing in ϑ and equals zero when ϑ=1/2. In the second case, for w∈[ϑ,1−1/n] we have $g_{n}\left(w\right)\leq \alpha \left(\vartheta \right)n+\beta_{n}\left(1-1/n\right)$. Again α(ϑ)<0 and $\beta_{n}\left(1-1/n\right)∼\frac{1}{2}\log n$ as n→∞, so that $g_{n}\left(w\right)$ is negative for w∈[ϑ,1−1/n] and sufficiently large n.□

## 3 THE ASYMMETRIC CASE

Proof of Proposition 1.6 ((asymmetric case) admitting Proposition 1.6) We fix $p\in (\frac{1}{2},1)$ and follow the steps from the symmetric case, but now we get instead of (25) the following coefficients in (24):

(48) $$a_{n}=\frac{\sqrt{n}}{z_{1,1}-z_{1,0}}\log\left(\frac{p}{1-p}\frac{e^{z_{1,1}/\sqrt{n}}-1}{1-e^{z_{1,0}/\sqrt{n}}}\right),$$

and

(49) $$b_{n}=n\log\left(\left(1-p\right)e^{-z_{1,0}a_{n}/\sqrt{n}}+pe^{-z_{1,1}a_{n}/\sqrt{n}}\right).$$

with $z_{1,0}=-\sqrt{p/(1-p)}$ and $z_{1,1}=\sqrt{(1-p)/p}$. Straightforward asymptotic expansions for n→∞ yield

(50) $$a_{n}=\frac{1}{2}-\frac{2p-1}{24\sqrt{p(1-p)}}n^{-\frac{1}{2}}+O\left(n^{-1}\right),$$

which slightly extends the result that is given in (Kreps and Schachermayer, 2020, Sec. 6), and

(51) $$b_{n}=\frac{1}{8}-\frac{1-p+p^{2}}{576p(1-p)}n^{-1}+O\left(n^{-2}\right).$$

Now we fix an arbitrary δ>0. For p∈(1/2,1) it follows from the asymptotics that

(52) $$0<a_{n}\leq \frac{1}{2},\;\frac{1}{8}-\delta \leq b_{n}\leq \frac{1}{8}$$

for all n sufficiently large. In fact, these inequalities are also true for p=1/2 as can be seen from (25).

Instead of (27) we now consider

(53) $$H_{y}^{n}\left(x\right)=V\left(y\exp\left(-a_{n}x-b_{n}\right)\right),\;x\in R.$$

If we are in the right tail and $z_{n,k}\geq 0$ then (52) yields $H_{y}^{n}\left(z_{n,k}\right)\leq H_{y}\left(z_{n,k}\right)$ and the uniform integrability follows just as in the symmetric case.

If we are in the left tail and $z_{n,k}\leq 0$ then (52) yields $H_{y}^{n}\left(z_{n,k}\right)\geq {\tilde{H}}_{y}\left(z_{n,k}\right)$, where ${\tilde{H}}_{y}\left(x\right)=V\left(\tilde{y}e^{-x/2-1/8}\right)$ with $\tilde{y}=ye^{\delta }$. Due to the convexity of V we have $v(\tilde{y})>-\infty$ and the uniform integrability follows just as in the symmetric case.□

Proof of Proposition 1.7 (asymmetric case) Following the steps from the symmetric case we now get

(54) $$\log f_{n,k}=\log\left(n!\right)-\log\left(k!\right)-\log\left(\left(n-k\right)!\right)+k\log p+\left(n-k\right)\log\left(1-p\right).$$

and

(55) $$\log\phi \left(z_{n,k+1}\right)=\frac{1}{2}\left(-\log\left(2\right)-\log\left(\pi \right)\right)-\frac{{\left(k-np+1\right)}^{2}}{2n(1-p)p}.$$

the key inequality (44) becomes

(56) $$\log\left(\frac{\sqrt{np(1-p)}f_{n,k}}{\phi (z_{n,k+1})}\right)\leq g_{n}\left(\frac{k}{n}\right),$$

with $g_{n}\left(w\right)=\alpha \left(w\right)n+\beta_{n}\left(w\right)$, where w∈[p,1], and

(57) $$\begin{array}{c} \alpha (w)=-w\log w-(1-w)\log(1-w)\\ +\frac{w^{2}}{2p(1-p)}+\left(\log\frac{p}{1-p}-\frac{1}{1-p}\right)w+\frac{p}{2(1-p)}+\log\left(1-p\right) \end{array}$$

and

(58) $$\beta_{n}\left(w\right)=-\frac{1}{2}\log\left(w(1-w)\right)+\frac{w}{p(1-p)}-\log2-\frac{1}{1-p}+\left(\frac{1}{12}+\frac{1}{2p(1-p)}\right)\frac{1}{n}.$$

Again we have $\alpha \left(p\right)=\alpha^{\prime }\left(p\right)=\alpha^{\prime \prime }\left(p\right)=0$. As regards the third derivative we find $\alpha^{\prime \prime \prime }\left(w\right)=\frac{1-2p}{{\left(p-1\right)}^{2}p^{2}}<0$ for w∈(p,1), and thus $\alpha^{\prime \prime \prime }\left(w\right)$, $\alpha^{\prime \prime }\left(w\right)$, $\alpha^{\prime }\left(w\right)$, and α(w) again are strictly negative and decreasing for w∈(p,1).

We have $\beta_{n}\left(p\right)=\frac{1}{12}\left(\frac{6}{np-np^{2}}+\frac{1}{n}-6\log\left(-4\left(p-1\right)p\right)\right)$ and $\beta_{n}^{\prime }\left(w\right)=\frac{1}{p-p^{2}}+\frac{1}{2-2w}-\frac{1}{2w}>0$ for w∈(p,1), thus $\beta_{n}\left(w\right)$ is strictly positive and strictly increasing for w∈(p,1).

Similarly as in the proof of Proposition 2.1, fix ϑ∈(p,1). For w∈[p,ϑ] we have $g_{n}\left(w\right)\leq \beta_{n}\left(\vartheta \right)$. As $\lim_{n\to \infty }\beta_{n}\left(\vartheta \right)=-1/2\log\left(\vartheta \left(1-\vartheta \right)\right)+\frac{h}{p(1-p)}-\frac{1}{1-p}-\log2$ it follows that $g_{n}\left(w\right)$ is bounded from above for the interval under consideration. For w∈[ϑ,1−1/n] we have $g_{n}\left(w\right)\leq \alpha \left(\vartheta \right)n+\beta_{n}\left(1-1/n\right)$. Now α(ϑ)<0 and $\beta_{n}\left(1-1/n\right)∼1/2\log n$ as n→∞ and thus $g_{n}\left(w\right)$ is negative for w∈[ϑ,1−1/n] and sufficiently large n.□

## Data Availability

Data sharing not applicable to this article as no datasets were generated or analyzed during the current study.

## References

[mafi12326-bib-0001] Abramowitz, M. , & Stegun, I. A. (Eds.) . (1992). Handbook of mathematical functions with formulas, graphs, and mathematical tables. New York: Dover Publications. Reprint of the 1972 edition.

[mafi12326-bib-0002] Arratia, R. , & Gordon, L . (1989). Tutorial on large deviations for the binomial distribution. Bulletin of Mathematical Biology, 51(1), 125–131. 2706397 10.1007/BF02458840

[mafi12326-bib-0003] Backhoff, J. , & Silva, F. J . (2018). Sensitivity analysis for expected utility maximization in incomplete Brownian market models. Mathematics and Financial Economics, 12(3), 387–411.

[mafi12326-bib-0004] Bahadur, R. R . (1960). Some approximations to the binomial distribution function. Annals of Mathematical Statistics, 31, 43–54.

[mafi12326-bib-0005] Bayraktar, E. , Dolinsky, Y. , & Guo, J . (2020). Continuity of utility maximization under weak convergence. Mathematics and Financial Economics, 14(4), 725–757.

[mafi12326-bib-0006] Bayraktar, E. , Dolinskyi, L. , & Dolinsky, Y . (2020). Extended weak convergence and utility maximisation with proportional transaction costs. Finance and Stochastics, 24(4), 1013–1034.

[mafi12326-bib-0007] Black, F. , & Scholes, M . (1973). The pricing of options and corporate liabilities. Journal of Political Economy, 81(3), 637–654.

[mafi12326-bib-0008] Cox, J. C. , & Huang, C.‐f . (1989). Optimal consumption and portfolio policies when asset prices follow a diffusion process. Journal of Economic Theory, 49(1), 33–83.

[mafi12326-bib-0009] Cox, J. C. , Ross, S. A. , & Rubinstein, M . (1979). Option pricing: A simplified approach. Journal of Financial Economics, 7(3), 229–263.

[mafi12326-bib-0010] Dalang, R. C. , Morton, A. , & Willinger, W . (1990). Equivalent martingale measures and no‐arbitrage in stochastic securities market models. Stochastics and Stochastics Reports, 29(2), 185–201.

[mafi12326-bib-0011] Desolneux, A. , Moisan, L. , & Morel, J.‐M . (2008). From Gestalt theory to image analysis. New York: Springer. A probabilistic approach.

[mafi12326-bib-0012] Dolinsky, Y. , & Neufeld, A . (2018). Super‐replication in fully incomplete markets. Mathematical Finance, 28(2), 483–515.

[mafi12326-bib-0013] Feller, W . (1945). On the normal approximation to the binomial distribution. Annals of Mathematical Statistics, 16, 319–329.

[mafi12326-bib-0014] Feller, W . (1968). An introduction to probability theory and its applications. (Vol. I. 3rd ed.). New York, London, Sydney: John Wiley & Sons, Inc.,.

[mafi12326-bib-0015] Harrison, J. M. , & Kreps, D. M . (1979). Martingales and arbitrage in multiperiod securities markets. Journal of Economic Theory, 20(3), 381–408.

[mafi12326-bib-0016] Harrison, J. M. , & Pliska, S. R . (1981). Martingales and stochastic integrals in the theory of continuous trading. Stochastic Processes and their Applications, 11(3), 215–260.

[mafi12326-bib-0017] He, H . (1991). Optimal consumption‐portfolio policies: A convergence from discrete to continuous time models. Journal of Economic Theory, 55(2), 340–363.

[mafi12326-bib-0018] Kambo, N. S. , & Kotz, S . (1966). On exponential bounds for binomial probabilities. Annals of the Institute of Statistical Mathematics, 18, 277–287.

[mafi12326-bib-0019] Karatzas, I. , Lehoczky, J. P. , & Shreve, S. E . (1987). Optimal portfolio and consumption decisions for a “small investor” on a finite horizon. SIAM Journal on Control and Optimization, 25(6), 1557–1586.

[mafi12326-bib-0020] Karatzas, I. , Lehoczky, J. P. , Shreve, S. E. , & Xu, G.‐L . (1991). Martingale and duality methods for utility maximization in an incomplete market. SIAM Journal on Control and Optimization, 29(3), 702–730.

[mafi12326-bib-0021] Krafft, O . (1969). A note on exponential bounds for binomial probabilities. Annals of the Institute of Statistical Mathematics, 21(1), 219–220.

[mafi12326-bib-0022] Kramkov, D. , & Schachermayer, W . (1999). The asymptotic elasticity of utility functions and optimal investment in incomplete markets. The Annals of Applied Probability, 9(3), 904–950.

[mafi12326-bib-0023] Kreps, D. M . (1981). Arbitrage and equilibrium in economies with infinitely many commodities. Journal of Mathematical Economics, 8(1), 15–35.

[mafi12326-bib-0024] Kreps, D. M . (2019). The Black‐Scholes‐Merton model as an idealization of discrete‐time economies. Cambridge: Cambridge University Press.

[mafi12326-bib-0025] Kreps, D. M. , & Schachermayer, W . (2020). Convergence of optimal expected utility for a sequence of discrete‐time markets. Mathematical Finance, 30(4), 1205–1228. 33041535 10.1111/mafi.12277 PMC7540176

[mafi12326-bib-0026] Kreps, D. M. , & Schachermayer, W . (2021). Asymptotic synthesis of contingent claims in a sequence of discrete‐time markets. Theoretical Economics, 16(1), 25–47.

[mafi12326-bib-0027] Littlewood, J. E . (1969). On the probability in the tail of a binomial distribution. Advances in Applied Probability, 1, 43–72.

[mafi12326-bib-0028] McKay, B. D . (1989). On Littlewood's estimate for the binomial distribution. Advances in Applied Probability, 21(2), 475–478.

[mafi12326-bib-0029] Merton, R. C . (1969). Lifetime portfolio selection under uncertainty: The continuous‐time case. The Review of Economics and Statistics, 51(3), 247–257.

[mafi12326-bib-0030] Merton, R. C . (1971). Optimum consumption and portfolio rules in a continuous‐time model. Journal of Economic Theory, 3(4), 373–413.

[mafi12326-bib-0031] Merton, R. C . (1973). Theory of rational option pricing. Bell Journal of Economics and Management Science, 4, 141–183.

[mafi12326-bib-0032] Mostovyi, O. , & Sîrbu, M . (2019). Sensitivity analysis of the utility maximisation problem with respect to model perturbations. Finance and Stochastics, 23(3), 595–640.

[mafi12326-bib-0033] Nagaev, S. V. , & Chebotarev, V. I . (2011). On the estimation of the closeness of the binomial distribution to the normal distribution. Rossiĭskaya Akademiya Nauk. Teoriya Veroyatnosteĭ i ee Primeneniya, 56(2), 248–278.

[mafi12326-bib-0034] Nagaev, S. V. , & Chebotarev, V. I . (2018). On large deviations for sums of i.i.d. Bernoulli random variables. Journal of Mathematical Sciences (New York), 234(6), 816–828.

[mafi12326-bib-0035] Nagaev, S. V. , Chebotarev, V. I. , & Zolotukhin, A. Y . (2016). A non‐uniform bound of the remainder term in the central limit theorem for Bernoulli random variables. Journal of Mathematical Sciences (New York), 214(1), 83–100.

[mafi12326-bib-0036] Okamoto, M . (1958). Some inequalities relating to the partial sum of binomial probabilities. Annals of the Institute of Statistical Mathematics, 10, 29–35.

[mafi12326-bib-0037] Prigent, J.‐L. (2003). Weak convergence of financial markets. Springer‐Verlag, Berlin.

[mafi12326-bib-0038] Rásonyi, M. , & Stettner, L . (2005). On utility maximization in discrete‐time financial market models. The Annals of Applied Probability, 15(2), 1367–1395.

[mafi12326-bib-0039] Short, M. (2013). Improved inequalities for the poisson and binomial distribution and upper tail quantile functions. *ISRN Probability and Statistics*, *2013*.

[mafi12326-bib-0040] Slud, E. V . (1977). Distribution inequalities for the binomial law. The Annals of Probability, 5(3), 404–412.

[mafi12326-bib-0041] Stanica, P. (2001). Good lower and upper bounds on binomial coefficients. JIPAM. Journal of Inequalities in Pure and Applied Mathematics, 2(3), Article 30, 5.

[mafi12326-bib-0042] van der Vaart, A. W . (1998). Asymptotic statistics, volume 3 of *Cambridge Series in Statistical and Probabilistic Mathematics*. Cambridge: Cambridge University Press.

[mafi12326-bib-0043] Young, D. M. , Turner, D. W. , & Seaman, Jr, J. W . (1988). A note on an exponential upper bound for binomial probabilities. The Texas Journal of Science, 40(3), 341–345.

[mafi12326-bib-0044] Zubkov, A. M. , & Serov, A. A . (2013). A complete proof of universal inequalities for the distribution function of the binomial law. Theory of Probability and its Applications, 57(3), 539–544.

